# Effectiveness of simplified predictive intubation difficulty score and thyromental height in head and neck surgeries: an observational study

**DOI:** 10.1016/j.bjane.2020.09.007

**Published:** 2020-09-30

**Authors:** Onur Selvi, Seda Tugce Kahraman, Serkan Tulgar, Ozgur Senturk, Talat Ercan Serifsoy, David Thomas, Ayse Surhan Cinar, Zeliha Ozer

**Affiliations:** a Maltepe University Faculty of Medicine, Istanbul, Turkey; b Sisli Hamidiye Etfal Training and Research Hospital, Istanbul, Turkey

**Keywords:** Difficult intubation, Difficult airway, Airway assessment, Intubação difícil, Via aérea difícil, Avaliação das vias aéreas

## Abstract

**Background and objectives:**

In this study, we aimed to investigate the predictive value of different airway assessment tools, including parts of the Simplified Predictive Intubation Difficulty Score (SPIDS), the SPIDS itself and the Thyromental Height Test (TMHT), in intubations defined as difficult by the Intubation Difficulty Score (IDS) in a group of patients who have head and neck pathologies.

**Methods:**

One hundred fifty-three patients who underwent head and neck surgeries were included in the study. The Modified Mallampati Test (MMT) result, Thyromental Distance (TMD), Ratio of the Height/Thyromental Distance (RHTMD), TMHT, maximum range of head and neck motion and mouth opening were measured. The SPIDSs were calculated, and the IDSs were determined.

**Results:**

A total of 25.4% of the patients had difficult intubations. SPIDS scores >10 had 86.27% sensitivity, 71.57% specificity and 91.2% Negative Predictive Value (NPV). The results of the Receiver Operating Curve (ROC) analysis for the airway screening tests and SPIDS revealed that the SPIDS had the highest area under the curve; however, it was statistically similar to other tests, except for the MMT.

**Conclusions:**

The current study demonstrates the practical use of the SPIDS in predicting intubation difficulty in patients with head and neck pathologies. The performance of the SPIDS in predicting airway difficulty was found to be as efficient as those of the other tests evaluated in this study. The SPIDS may be considered a comprehensive, detailed tool for predicting airway difficulty.

## Resumo

**Justificativa e objetivos:**

Neste estudo, avaliamos o valor preditivo de diferentes ferramentas de avaliação das vias aéreas, incluindo componentes do Escore Simplificado Preditivo de Intubação Difícil (ESPID), o próprio ESPID e a Medida da Altura Tireomentoniana (MATM), em intubações definidas como difícies pelo Escore de Dificuldade de Intubação (EDI) em um grupo de pacientes com patologia de cabeça e pescoço.

**Método:**

Incluímos no estudo 153 pacientes submetidos a cirurgia de cabeça e pescoço. Coletamos os resultados do Teste de Mallampati Modificado (TMM), Distância Tireomentoniana (DTM), Razão Altura/Distância Tireomentoniana (RADTM), MATM, amplitude máxima de movimentação da cabeça e pescoço e da abertura da boca. Os ESPIDs foram calculados e os EDIs determinados.

**Resultados:**

Observamos intubação difícil em 25,4% dos pacientes. Os escores de ESPID > 10 tiveram sensibilidade de 86,27%, especificidade de 71,57% e valor preditivo negativo de 91,2% (VPN). O resultado da análise da curva de operação do receptor (curva ROC) para os testes de avaliação das vias aéreas e ESPID mostrou que o ESPID tinha a maior área sob a curva; no entanto, foi estatisticamente semelhante a outros testes, exceto para o TMM.

**Conclusões:**

O presente estudo demonstra o uso prático do ESPID na previsão da dificuldade de intubação em pacientes com patologia de cabeça e pescoço. O desempenho do ESPID na predição de via aérea difícil mostrou-se tão eficiente quanto os demais testes avaliados neste estudo. O ESPID pode ser considerado ferramenta abrangente e detalhada para prever via aérea difícil.

## Introduction

Difficult intubations may increase anesthetic-related morbidity and mortality rates and has been reported to be observed in 0.5% to 10% of patients.^1^ The proportion of unanticipated difficult intubations in daily clinical practice ranges from 75% to 93%, which emphasizes the extent to which airway management difficulty is not predicted.^2^ Existing head and neck pathologies and other risk factors leading to difficult intubations may hinder a successful intubation. The incidence of difficult intubations is reported to be higher in patients with head and neck pathologies than in the normal population.^3^ Maintaining airway safety in this patient group is challenging, and failure to do so may lead to fatal consequences.^4^ It is therefore of utmost importance for an anesthesiologist in clinical practice to predict airway difficulty. However, there is no correlation between what is known and what is applied in clinical practice, and airway assessment is highly dependent on the judgment of an individual anesthesiologist.^5^ This is why there is ongoing research to identify a practical airway evaluation method that is not time consuming and has high interrater reliability. Etezadi et al. suggested that the Thyromental Height Test (TMHT) is a single reliable test with high predictive value.^6^ However, no single measurement is accepted as a gold standard test since several airway elements affect the difficulty level of an airway. The American Society of Anesthesiology (ASA) suggests that multiple airway features should be considered when evaluating airways preoperatively.^7^ Combinations of several airway features and measurements have been used to develop multivariate risk indexes for the prediction of intubation difficulty.^8^ The Simplified Predictive Intubation Difficulty Score (SPIDS) is one of the several proposed multivariate tests.^7^

Herein, we aimed to measure the predictive values of the SPIDS and assess the usefulness of the TMHT in anticipating airway difficulty.

## Methods

This prospective, observational study was performed between May 2016 and December 2017 at the Maltepe University Faculty of Medicine and Sisli Hamidiye Etfal Training and Research Hospital. Institutional Ethics Committee approval was obtained prior to the commencement of the study, and written informed consent was obtained during the preanesthesia visit for the inclusion of data in this study. The study has been registered at www.Clinicaltrials.gov with the ID number NCT03320278. Strengthening the Reporting of Observational Studies in Epidemiology (STROBE) Guidelines were followed and implicated in this observational study. All patients from plastic surgery and Ear, Nose & Throat (ENT) clinics who underwent surgical procedures for head and neck pathologies were included in the study in the time frame in which it was conducted. A list of the operations examined in this study is provided in Table 1. Patients who were assessed by only trained personnel were invited to participate in the study. Patients who were aged under 18, did not provide consent, underwent an emergency procedure, or were undergoing nasal intubation or awake fiberoptic intubations were excluded from the study. Patients who received different drugs by induction rather than by the methods in the predetermined protocol were excluded. Patients who underwent planned tracheostomy, predetermined videolaryngoscopy, and laryngeal masks were also excluded. The files of 7 patients were considered to have missing data due to a discrepancy in the case forms or missing information.

**Table 1:** List of surgical procedures and pathologies.

Thyroid surgery	35
Neck dissection	28
Laryngectomy	18
Neck mass tumor surgery	22
Tounge tumor surgery	12
Maxillofacial fracture	28
Submandibular mass	5
Nasopharynx tumor	2
Vocal cord polyp	2
Endolaryngeal tumor	1

Total	153

### Outcomes

For the primary outcome measure, we aimed to assess the performance of the SPIDS in predicting intubation difficulty in a group of patients who have head and neck pathologies and therefore are more likely have a difficult airway. For the secondary outcome measure, we aimed to compare the predictive ability of the TMHT for intubation difficulty with that of the other single assessment tests included in the SPIDS. The Intubation Difficulty Score (IDS) was the index we used to determine intubation difficulty. A total score higher than five in the IDS indicates a difficult intubation.

### Measurements

During the preanesthesia evaluation of the patients, their height, age, weight, ASA scores, Mallampati scores, measurements of the head and neck movement angles and any associated airway pathology associated with difficult intubations were noted on the preanesthesia evaluation forms. Patients’ thyromental distance, thyromental height, and mouth opening values were measured with a depth gauge (ASIMETO® Electronic Depth Gage, 0–6″/0–150 mm) digitally in the preoperative waiting room by trained anesthesia nurses.

In the operation room, the intubations were conducted by one of the two participating anesthesiologists. An appropriate blade size was selected in concordance with the patient's size and height. The majority of the intubations were completed with no. 4 Macintosh blade. Usage of no. 5 Macintosh blade was preferred only for a few patients who were either very tall, overweight or both, to prevent misevaluation of the airway that may have affected overall IDS. This decision was based on the comparison of the distance from the midline of the upper incisor teeth to the angle of the mandible with the length of the selected Macintosh blade. Following the contour of the face, the tip of the blade was extended toward the anatomical landmark, which was the angle of the mandible. A no. 5 Macintosh blade was chosen when the length of a no. 4 Macintosh blade was inadequately short according to this measurement.

All difficult airway equipment was readily prepared in advance and standard difficult airway guidelines were followed when the airway of a patient was determined to require difficult intubation. A McGRATH® (Aircraft Medical, UK) portable video laryngoscope was used as the back-up plan in difficult intubations. None of the patients had failed endotracheal intubation.

After the intubation, the anesthesiologists provided Cormack-Lehane (C-L) scores and calculated the information needed to complete the IDS to determine the intubation difficulty with a standardized method. Muscle relaxation was provided with 0.6 mg kg^−1^ rocuronium bromide. Finally, all scores were recorded in the patient's study forms.

#### Modified Mallampati Test (MMT)

This test has four grades, so the score can be between 1 and 4 points. Mallampati scores of 3 and 4 are considered predictive of a difficult intubation. Patients are asked to make an “a” sound without phonation while opening the mouth, and the pharyngeal structures are visualized with the head in slight extension.^9^

#### Thyromental distance (TMD)

The head is fully extended, and the distance between lower border of the mandibular mentum and thyroid protrusion is measured along a straight line. The short thyromental distance (TMD ≤6.5 cm) has been correlated with difficult direct laryngoscopic intubations in adult patients.^9^

#### Ratio of Height/Thyromental distance (RHTMD)

The ratio of height in cm and thyromental distance (cm) is calculated. A RHTMD ≥25 is considered one of the risk factors in the SPIDS.^7^

#### Thyromental Height Test (TMHT)

This technique was proposed by Etzadi et al. for predicting intubation difficulty, and a thyromental height less than 50 mm is considered difficult. It is considered a warning signal for an existing difficult intubation. The distance between the anterior border of the mentum and the anterior border of the thyroid cartilage is measured with a digital depth gauge. The patient is positioned in the supine position, and the mouth should be closed.^6^

#### Intubation Difficulty Score (IDS)

The IDS include seven parameters, resulting in a progressive, quantitative determination of intubation complexity (Table 2). The IDS is calculated immediately after intubation. The score might then be used to compare the difficulty of the intubation under varying circumstances by isolating variables of interest. A total score that is higher than 5 corresponds to a difficult intubation.^7^

**Table 2:** Intubation difficulty score (IDS).

Parameters	Score
Number of attempts > 1	Each 1 point
Number of operators > 1	Each 1 point
Number of alternative techniques	Each 1 point
Cormack-Lehane grade	Grade1 = 0 point
	Grade 2 = 1 point
	Grade 3 = 2 point
	Grade 4 = 3 point
Lifting Force	Normal = 0 point
	Increased = 1 point
Laryngeal pressure	Not applied = 0 point
	Applied = 1 point
Vocal cord mobility	Abduction = 0 point
	Adduction = 1 point
Total score = sum of scores	IDS > 5 Moderate to difficult intubation

IDS, Intubation Difficulty Score.

#### The Simplified Descriptive Intubation Difficulty Score (SPIDS)

The details of the SPIDS are provided in Table 3. The maximum score can be 55, and a total score <10 corresponds to a difficult intubation. For the calculation of the SPIDS score, the following parameters are needed.^7^

1- History of pathologies or existing problems that might be related to a difficult intubation, such as obstructive sleep apnea, facial malformations, and cervical dislocation, are scored as “yes” or “no”.
2- Mouth opening: The patient is asked to open the mouth fully and the maximum interincisor gap or, for edentulous patients, the intergingival gap is measured in cm. The cut-off value is set to be 3.5 cm.
3- Maximum range of motion of the head and neck measurement: Patients are asked to fully flex and to fully extend their head and neck. The angle between the bridge of the nose in flexion and in extension is measured with an angle meter.
4- Modified Mallampati Test. All these airway assessment tests were conducted in the preoperative visit and preoperative period by experienced and trained staff.

**Table 3:** The Simplified Descriptive Intubation Score (SPIDS).

Risk factors	Points of the “simplified scores”
*Pathologies associated with difficult intubation*	
No	0
Yes	10

*Mouth opening*	
≥ 3.5 cm	0
<3.5 cm	10

*RHTMD*	
<25 cm	0
≥25 cm	10

*Maximum range of head and neck movement*	
≥80°	0
<80°	5

*MMT*	
Class 1	0
Class 2	10
Class 3	15
Class 4	25
Total possible	55

RHTMD, Ratio of Height Thyromental Distance; MMT, Modified Mallampati Test.

To prevent bias, four senior anesthesiologists with a minimum of seven years of experience and two anesthesia nurses participated in the study. The anesthesiologist who performed the intubations was blinded to the results of the preoperative measurement tests, except for the Mallampati score and angles of head and neck movements. All intubations were performed three minutes after the application of a neuromuscular block. Guide wires were attached to all of the intubation tubes. Additionally, anesthesia nurses were educated on how to take the appropriate airway measurements and fill in the study forms before the commencement of the study.

### Statistics

The minimum sample size calculation was conducted for comparisons of ROC curves and 126 patients were required to detect at least 0.200 difference between areas under the ROC curves, as for alpha = 0.05 and 1-beta = 0.80 levels. A total of 153 eligible patients were consecutively enrolled in this observational study during the time frame in which the study was conducted. Patient records with inconsistencies and incomplete data were eliminated before statistical study began. Statistical analyses were performed by using NCSS (Number Cruncher Statistical System). Descriptive data were presented using the mean, median, first Quartile (Q1), third Quartile (Q3), frequency, rate, minimum and maximum values. Comparisons of the two groups’ parameters showing normal distributions were analyzed by using Student's *t*-tests. The Mann-Whitney *U* test was used for the parameters that did not follow a normal distribution. The cut-off values for the parameters were determined by assessing the sensitivity, specificity, Positive Predictive Value (PPV), Negative Predictive Value (NPV), accuracy, Youden Index and Odds Ratio (OR). Receiver-Operating Characteristic (ROC) analysis and curves were conducted to determine and illustrate the diagnostic ability of the parameters. The DeLong method was used for the comparison of the Area under the Receiver Operation Characteristic (AuROC). The *p*-value <0.05 was considered statistically significant.

## Results

A total of 413 potentially eligible patients initially were assessed for the study. One hundred fifty-three patients (45 women, 108 men, aged 19–85) were finally included in the study. The flow diagram discloses the information regarding study recruitment and missing data. The demographic data and characteristics of the participants are shown in Table 4. Thirty-nine (25.0%) patients were considered to require a difficult intubation due to an IDS score >5. Table 5 presents the relationship between the demographic data and difficult intubations (IDS > 5). The patients in the difficult intubation group had a higher ASA score, weight, height, Body Mass Index (BMI) and age compared to easy intubation group (*p* < 0.01, *p* < 0.01, *p* < 0.01, *p* < 0.05, *p* < 0.05, respectively).

**Table 4:** Characteristics and demographic data of the patients.

Number of patients according to ASA	*n*
I	65
II	62
III	23
IV	3

	Mean ± SD (Min–Max)
Age (years)	48.6 ± 15.6 (19–85)
Weight (kg)	74.2 ± 13.6 (39–107)
Height (cm)	168.3 ± 7.1 (152–190)
BMI (kg m^-2^)	26.20 ± 4.78 (14.35–45.45)

ASA, American Society of Anesthesiology; BMI, Body Mass Index.

**Table 5:** Demographic data and difficult intubation (IDS > 5).

	Difficult intubation IDS		*p*
	IDS ≤ 5 (*n* = 114)	IDS > 5 (*n* = 39)	
	Mean ± SD	Mean ± SD	
Age (years)	46.30 ± 16.2	55.3 ± 11.40	0.00172^a^^.^^c^
Weight (kg)	71.9 ± 12.8	80.7 ± 13.8	0.00037^a^^,^^d^
Height (cm)	169.0 ± 7.04	166.0 ± 7.00	0.03584^a^^,^^c^
BMI (kg m^−2^)	25.5 ± 4.18	29.26 ± 5.15	0.00001^b^^,^^d^
ASA (Median)	1.61 ± 0.72 (1.00)	2.21 ± 0.77 (2.00)	0.00003^b^^,^^d^

ASA, American Society of Anesthesiology; BMI, Body Mass Index.

a Student *t*-Test.

b Mann Whitney *U* Test.

c *p* < 0.05

d *p* < 0.01.

The comparisons of the TMHT, TMD, RHTMD, SPIDS, and IDS according to intubation difficulty are shown in Table 6.

**Table 6:** Comparison of TMH, TMD, RHTMD, SPIDS, and IDS according to difficulty in intubation.

Test	IDS ≤ 5 (*n* = 114) Median (Q1, Q3)	IDS > 5 (*n* = 39) Median (Q1, Q3)	*p*
TMD	8.62 (7.75–9.4)	6.38 (5.9–7.21)	<0.001^a^
Height/TMD	20.06 (17.74–22.14)	25.66 (23.36–27.2)	<0.001^a^
TMH	5.2 (4.7–5.72)	3.81 (3.3–4.32)	<0.001^a^
SPIDS	10 (0–15)	30 (25–50)	<0.001^a^

RHTMD, Ratio of Height Thyromental Distance; SPIDS, Simplified Predictive Intubation Difficulty Score, IDS, Intubation Difficulty Score; TMD, Thyromental Distance; TMH, Thyromental Height.

Mann-Whitney *U* test.

a *p* < 0.01.

**Figure 1:**
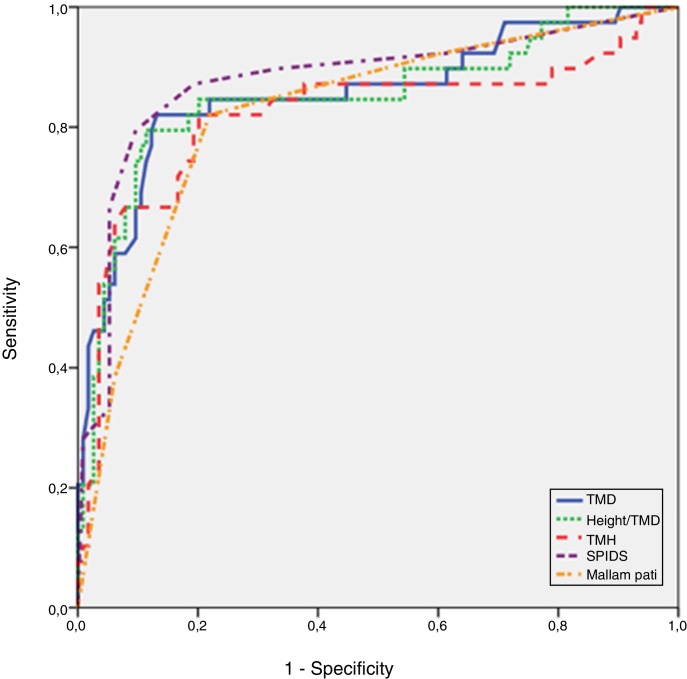
Receiver Operating Curve (ROC) of airway screening tests and SPIDS.

**Figure 2:**
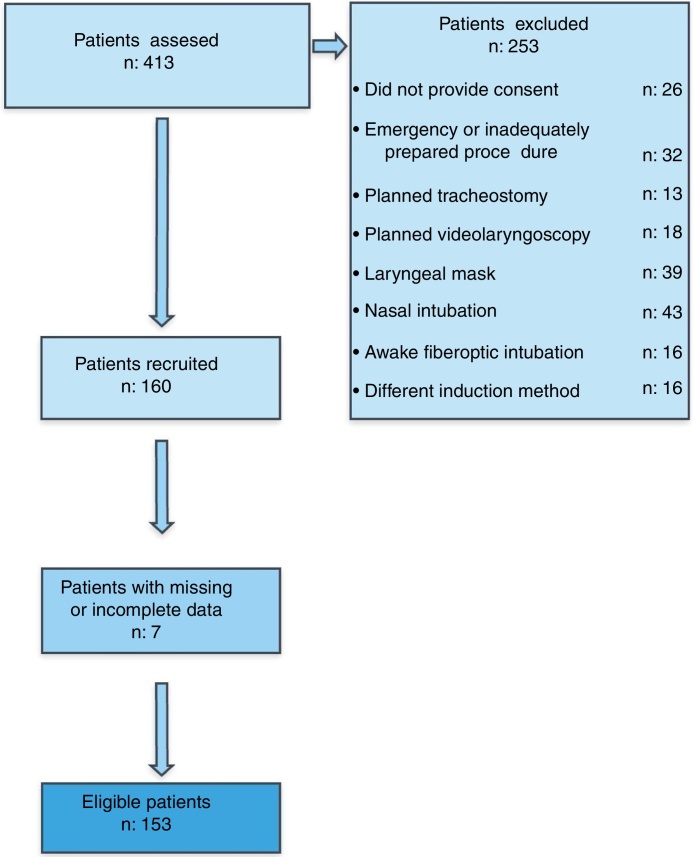
Patient recruitment flow diagram.

The diagnostic screening test results and ROC curve analysis results, including the sensitivity, specificity, PPV and NPV values for different cut-off values for the SPIDS, TMH, TMD, RHTMD and Mallampati >2, are shown in Table 7. The results of the ROC analysis for the airway screening tests and SPIDS are shown in Table 8 and Fig. 1. The comparison of the Area Under the Curve (AUC) between each test is presented in Table 9 (Fig. 2).

**Table 7:** Statistical results for the diagnostic tests and SPIDS to predict difficult intubations according to the IDS.

Test	Cut-off value	Sensitivity (95% CI)	Specificity (95% CI)	PPV (95% CI)	NPV (95% CI)	Accuracy	Youden index	Odds Ratio
TMD	≤7.27	82.05 (66.5–92.5)	86.84 (79.2–92.4)	68.1 (52.9–80.9)	93.4 (86.9–97.3)	0.856	0.689	30.171
TMD	≤6.5	41.18 (27.6–55.8)	96.08 (90.3–98.9)	84.0 (63.9–95.5)	76.6 (68.3–83.6)	0.778	0.373	22.947
Height/TMD	>23.25	79.49 (63.5–90.7)	88.60 (81.3–93.8)	70.5 (54.8–83.2)	92.7 (86.0–96.8)	0.863	0.681	27.679
Height/TMD	≥25	47.06 (32.9–61.5)	95.10 (88.9–98.4)	82.8 (64.2–94.2)	78.2 (69.9–85.1)	0.791	0.422	19.782
TMH	≤4.38	82.05 (66.5–92.5)	79.82 (71.3–86.8)	58.2 (44.1–71.3)	92.9 (85.8–97.1)	0.804	0.619	18.087
TMH	<5	80.39 (66.9–90.2)	64.71 (54.6–73.9)	53.2 (41.5–64.7)	86.8 (77.1–93.5)	0.699	0.451	11.228
SPIDS	>20	79.49 (63.5–90.7)	90.35 (83.4–95.1)	73.8 (58.0–86.1)	92.8 (86.3–96.8)	0.876	0.698	36.284
SPIDS	>10	86.27 (73.7–94.3)	71.57 (61.8–80.1)	60.3 (48.1–71.5)	91.2 (82.8–96.4)	0.765	0.578	17.500
MMT	>2	82.05 (66.5–92.5)	78.07 (69.4–85.3)	56.1 (42.4–69.3)	92.7 (85.6–97.0)	0.791	0.601	16.274

SPIDS, Simplified Predictive Intubation Difficulty Score; IDS, Intubation Difficulty Score; TMD, Thyromental Distance; TMH, Thyromental Height; MMT, Modified Mallampati Test; PPV, Positive Predictive Value; NPV, Negative Predictive Value.

**Table 8:** Results of Receiver Operating Curve (ROC) analysis for airway screening tests and SPIDS.

Test	Area	Standard error	95% CI	*p*
TMD	0.856	0.040	0.791, 0.908	<0.001^a^
Height/TMD	0.851	0.041	0.784, 0.903	<0.001^a^
TMH	0.820	0.058	0.750, 0.878	<0.001^a^
SPIDS	0.878	0.038	0.815, 0.925	<0.001^a^
MMT	0.823	0.039	0.753, 0.880	<0.001^a^

SPIDS, Simplified Predictive Intubation Difficulty Score; TMD, Thyromental Distance; TMH, Thyromental Height; MMT, Modified Mallampati Test.

a *p* < 0.01.

**Table 9:** The comparison of Area Under Curve (AUC) of Diagnostic Tests.

Tests	Area difference	Standard error	*p*-Value
TMD – Height/TMD	0.005	0.010	0.597
TMD – TMH	0.036	0.025	0.142
TMD – SPIDS	0.022	0.046	0.638
TMD – MMT	0.033	0.049	0.498
Height/TMD – TMH	0.031	0.026	0.242
Height/TMD – SPIDS	0.027	0.044	0.538
Height/TMD – MMT	0.028	0.048	0.563
TMH – SPIDS	0.058	0.055	0.292
TMH – MMT	0.003	0.055	0.959
SPIDS – MMT	0.055	0.026	0.037^a^

SPIDS, Simplified Predictive Intubation Difficulty Score; TMD, Thyromental Distance; TMH, Thyromental Height; MMT, Modified Mallampati Test.

a *p* ≤ 0.05 was considered statistically significant.-

## Discussion

This prospective study, conducted in a group of patients with head and neck pathologies, revealed that the SPIDS shows the highest performance as a predictive index compared to other airway assessment tools. The SPIDS demonstrated a significantly higher performance (86% sensitivity, 60% PPV) in this study compared to that in the original study in which this technique was first reported (65% sensitivity, 14% PPV). In patients with head and neck pathologies, the SPIDS was able to correctly identify more patients who truly had difficult intubations. A third of our study population consisted of patients with one or more pathologies related to difficult intubations. We believe that our results are improved compared to those in the original study, which included the general population, due to the demographics of the patients included in this study.^7^

The SPIDS was designed to identify risk factors for difficult intubations by using a “weighted score” based on adjusted risk factors predicting intubation difficulty. The SPIDS consists of four different measurements: mouth opening, the TMD, maximum head and neck movements and the Mallampati score.^7^ Since most of these tests are already evaluated as a part of our daily pre-assessment visit, the overall time spent on the SPIDS did not significantly affect the time patients spent in our clinical practice. However, inevitably, it requires more time to complete the whole test.

We chose to evaluate this test in patients with head and neck pathologies because the SPIDS was developed by using the reference risk index developed by Arne et al.^10^ This method was developed and validated first in ENT and general surgery patients. Additionally, we believe that ENT patients and patients with upper airway pathologies might be the most appropriate group of patients to evaluate the accuracy of the SPIDS due to the high risk of difficulties during airway management. Forty percent of difficult airway cases in the 4th National Audit Project (NAP4) were related to diseases involving the head, neck or trachea, and unfortunately, 70% of these cases resulted in airway obstruction.^11^ In patients with head and neck pathologies, emergency surgical procedures for airway access might be the only method of treatment in when intubation fails. In some cases, even this procedure may not prevent life-threatening airway complications.^4^ Therefore, examining SPIDS in this group of patients was reasonable and worthwhile.

The SPIDS is superior to single measurement tests because it necessitates communication between the examiner and the patient and combines some single airway tests together. The questions posed by the SPIDS lead to the identification of pathologies or existing problems that might be related to difficult intubations, such as obstructive sleep apnea, facial malformations, and cervical dislocation. The American Society of Anesthesiology (ASA) also recommends a preoperative assessment of the patient's airway based on 11 anatomical variables and combining airway risk factors for difficult intubations undoubtedly results in higher diagnostic accuracy.^5^, ^12^ However, while combining two or more screening tests can have higher positive predictive value, this accuracy is accomplished at the cost of reduced sensitivity and a higher incidence of false negative predictions.^8^ A false negative result may expose patients to increased perioperative risks and hypoxia, and a false positive result may lead to unnecessary procedures and less cost-effective alternative techniques being performed. In a recent review that evaluated bedside screening tests in 133 studies with 844,206 participants, these tests were found to be inconvenient for detecting unanticipated difficult airways since difficult airways were not detected in a large number of people who had a difficult airway.^13^

While the TMHT and other airway examination tests evaluated in this study as a part of the SPIDS showed no statistical advantage over the others, it cannot be said that these are strong, reliable methods in predicting intubation difficulty. In these tests, the TMD is a widely studied assessment tool, and a TMD ≤6.5 cm is considered a risk factor in the SPIDS. In this study, the AUC results showed that the TMD is an effective tool in other airway assessment tests (OR = 22.9). However, different studies have reported the use of different cut-off values for this controversial test, ranging from 6 cm to 8 cm.^9^ Therefore, another method, the RHTMD, which uses the patient's height and body proportions together with the TMD, was proposed. Several studies have compared the RHTMD with other single airway assessment tests, and it has been reported to be a better single predictor test with a cut-off value of ≥23.5.^14^, ^15^ Our study showed 47% sensitivity, 95% specificity, 83% PPV and 78% NPV for the RHTMD, which were lower than the corresponding results in previous studies. We believe this is due to an RHTMD ≥25 rather than an RHTMD ≥23.5 being considered a risk factor in the SPIDS. The AUC analyses in our study showed that the RHTMD is only as efficient as the other single assessment tests.

The TMHT was also evaluated in this study, as it has previously been proposed in several studies as a promising single airway test with high sensitivity and specificity based on airway examinations.^16^ This test was first proposed by Etezadi et al.^6^ A TMHT shorter than 5 cm may indicate an excessively caudal and anterior larynx, which is correlated with difficult laryngoscopy. However, other airway pathologies causing distortions or narrowing may go undiagnosed with this test. Similar to other single airway assessment tests, the TMHT does not elucidate low airway pathologies. The TMHT is also not useful for the evaluation of the dynamic structures of central airway changes that are related to posture and breathing.^17^ We examined a highly selective population with head and neck pathologies; therefore, we could not replicate the originally described efficacy of the TMHT in this study.

When all tests were evaluated, the AUC comparison revealed that the only statistically significant difference was between the SPIDS and Modified Mallampati Tests. However, it should be noted that these two models have different methodologies and therefore predict airway difficulty in different ways. The SPIDS is a multivariate test, and the Modified Mallampati Test is a single assessment tool. In this study, a Mallampati score >2 showed 82% sensitivity, 78% specificity, 56% PPV and 93% NPV. However, although it is well integrated in clinical practice and a widely used test in Europe and North America, we are not able to recommend the Modified Mallampati Test as a stand-alone tool. The Modified Mallampati Test addresses only a limited part of the overall assessment of the airway, and there is substantial variability in the reported accuracy among the studies with poor discriminative power when the test is used alone.^18^

In this study, 50 patients had an SPIDS score of 10 points due to pathological conditions associated with difficult intubations. The majority of the pathological conditions associated with difficult intubations included facial bone fractures, neck masses, large thyroid tissues, deviated tracheas, obstructive sleep apnea syndrome, soft tissue stiffness due to diabetes mellitus or rheumatologic diseases, ankylosing spondylitis, cervical disc hernia, cervical rheumatism, oral cavity masses, vocal cord masses and tongue tumors. In patients with these pathologies and additional risk factors leading to a SPIDS score >10, the odds ratio of observing a difficult intubation is calculated to be 17.5. The odds ratio rises to 36.2 when the SPIDS score is >20. This proves that a proper airway evaluation may be incomplete without an assessment of the old anesthetic records.^18^ Thus, questioning the patient's own experience with previous surgical interventions requiring airway management should be considered an important aspect of airway evaluation which is a mandatory element of SPIDS.

The relationship between multiple airway measures and difficult intubations has been previously evaluated, and several models have been proposed. Considerable disagreement in the assessments of the risk factors may decrease the value of these models.^5^, ^8^, ^10^ The “Simplified Airway Risk Index” (SARI) model is one of these models and was first described by El-Ganzouri et al.^8^ In a study that included 26 departments and 64273 participants from Denmark, no significant difference was found in the predictive accuracy between centers using SARI and those who were not.^19^ To our knowledge, the SPIDS has not been compared to other single airway assessment tests, and this study performed this comparison.

An airway assessment test is also expected to be practical and useful.^9^ Therefore, in our daily clinical practice, using the SPIDS should not have a negative effect on time management. However, the calculation of the total score may appear to be protracted, time-consuming and complicated due to the need to evaluate several aspects of an airway. Although we did not measure the required mean time for using SPIDS, hypothetically, a possible delay caused by SPIDS may hinder the integration of this test into clinical use. An airway assessment test should have high sensitivity and specificity and minimal false positive and false negative values. The SPIDS slightly improved the predictive accuracy of preoperative airway assessment and its performance in predicting airway difficulty was found to be as efficient as those of the other tests evaluated in this study. But it is important that in SPIDS, a total score strictly above 10 obligates the anesthesiologist to plan for a difficult airway management strategy. Providing the anesthesiologist with a numeric score in case of an existing difficult airway can be considered a highlight of SPIDS. This numeric score may reduce the subjective evaluation of the airway and the plan of the airway management and may even reduce adverse events. Having a judiciously decided airway management plan before starting the induction of anesthesia may decrease airway related morbidity and mortality. Unfortunately, we did not evaluate the clinical benefits of using SPIDS in this study. This information may be examined in future studies.

### Limitations of the study

The most challenging part of this study was recruiting a large number of patients with head and neck pathologies. We chose to focus only on head and neck surgeries, leading to a small number of patients evaluated. Undoubtedly, assessing the SPIDS in a more homogenous group would have been more ideal. However, despite the limited number of patients, we were able to overcome our main limitation. Therefore, determining the adequacy of the SPIDS in predicting airway difficulty in a limited time frame was only feasible in a heterogeneous group. The small number of patients we enrolled in this study prevented us from being able to generalize the results to the general population. The SPIDS should be examined in other at-risk groups, such as pregnant or obese individuals.

SPIDS apparently demands more time than any simple airway measurement test. However, we did not measure the time required to implement the SPIDS and this should be considered as a limitation.

Neuromuscular monitoring and muscle relaxation measurements were not routinely performed, and sufficient levels of muscle relaxation were not confirmed with any device to standardize the effect of the neuromuscular blocking agent. Nonobjective monitoring of the neuromuscular block can distort Cormack-Lehane visualization. To standardize the level of muscle relaxation, an equal amount of neuromuscular blocking drug per kg of the patient's weight was administered, and each patient was intubated after two and a half minutes. Cross-checking of the Cormack-Lehane grading and IDS could have provided more accurate results, but the limited number of trained staff for the study prevented us from performing these steps. However, we recruited investigators with a minimum of seven years of experience in anesthesia to decrease interobserver variability.

### Future insights

The development of new technologies and more advanced methods such as the Ultrasound (US) imaging assessment of the airway, 3D printing and dynamic CT scans may have the potential to provide comprehensive knowledge of an airway.^17^, ^20^ This study, however, aimed to assess the ability of the SPIDS and some other conventional airway assessment tests in predicting intubation difficulty. Although recent advances in airway assessment methods have been included in difficult airway situations, they may not replace the tests examined in this study due to their complexity and availability.

## Conclusion

The SPIDS has been found to be as efficient as the other single airway assessment tests examined in this study and it also provides us with a useful numeric score. Additionally, when compared to other conventional single airway assessment tests, the SPIDS examines and evaluates different aspects and features of an airway in an organized and guided manner. Therefore, although it showed no superiority to the other tests, the SPIDS might be an alternative approach that is helpful for improving our ability to predict airway difficulty. The SPIDS may be a comprehensive, noninvasive, with no procedural costs, although it is time-consuming and complex. However, it is clear that we are still in need of additional technologies for more accurate predictions of a difficult airway and a safer anesthesia practice.

## Conflicts of interest

The authors declare no conflicts of interest.
